# Lipidomics: a promising cancer biomarker

**DOI:** 10.1186/s40169-018-0199-0

**Published:** 2018-07-30

**Authors:** Furong Yan, Hong Zhao, Yiming Zeng

**Affiliations:** Department of Pulmonary and Critical Care Medicine, The Second Affiliated Hospital of Fujian Medical University, Quanzhou, Fujian Province China

**Keywords:** Clinical lipidomics, Cancer, Biomarkers, Early diagnosis, Individualized treatment

## Abstract

The prevention, diagnosis and targeted therapies of cancer are important in cancer controlling and treatment. The present challenge about cancer biomarker still remains in identifying the special biomarkers for predicting cancer risk and assessing patient’s response during anticancer treatment. Lipidomics, in simple definition is the quantification of all lipids in a confined biological entity. Lipids play roles in membrane structure, energy storage, and signal transduction as well as in human cancers. Previous researches indicated that lipids may serve as a promising biomarker in the early diagnoses and individualized treatment of cancer.

Metabolomics is a rapid developing area to provide valuable information about diseases, of which a subset is lipidomics defined as a study about the content and function of whole lipids in the cell or tissue in biologic systems. Lipids are crucial in many cellular processes, including cell survival, proliferation, interaction, and apoptosis. Lipids consist of cholesterol, glycerol-based phospholipids, and ceramide-based sphingolipids. Dysfunction of lipids metabolism was found to be associated with pathogenesis of many diseases [1], such as diabetes, Alzheimer’s disease, hypertension, and cancers. Lipidomics is the quantitative of lipids determined in cell, tissue, body fluid, or organism at a schedule time, as an emerging field about the systemic analysis of lipids and their metabolites and interactions. Studies on lipidomics suggested that lipids play many important functions in living organisms, especially the transformation, progression, and metastasis of cancer [2]. Some cancer cells were found to stay alive through energy from fatty acid oxidation. Several studies found many lipids increased in cancer, such as the lysophospholipids in ovarian cancer, glycerophospholipids in hepatocellular carcinoma, acylcarnitines, glycerophospholipids and arginine in prostate cancer, sphingolipid 1-phosphate in ovarian or glioma and breast cancer [3–8]. Studies also suggested the choline-containing lipids and phospholipids could increase during the metastasis of cancer cells.

An growing evidence demonstrates that lipid metabolism is strongly related to colorectal cancer. Most of high serum triglycerides were found to change on colorectal adenoma [9]. The serum levels of total cholesterol, lipoprotein cholesterol, apolipoprotein A1, and apolipoprotein B reduced in patients with colorectal cancer, while the level of free cholesterol increased [9]. Therefore, lipidomics is proposed as a viable method to monitor the prognosis, diagnosis and treatment of cancer and acts as a new cancer biomarkers. Lv et al. recently published an initial clinical study on the profiles of plasma lipidome between health and patients with lung cancer or among patients with squamous cell carcinomas, adenocarcinoma, or small cell lung cancer [10]. This is the first time to study the heterogeneity of lipidomic profiles among lung cancer subtypes of patients by integrating lipidomic data with lipid protein-associated genomic expression among lung cancer subtypes. Although the study was presented as a short communication and lacked details of analyses across lipidomic data and lipid-protein-associated genomic data, a large number of lipid protein-associated genes significantly changes among cancer subtypes, with correlations with altered species and spatial structures of lipid metabolites. It would be more valuable if those lipidomic and genomic data can be fused and integrated with clinical phenomes described as clinical trans-omics [11]. The question is whether lipidomic profiles are sensitive and specific enough to identify the heterogeneity among cancer subtypes, stable and repeatable enough to link with alterations of clinical phenomes and patient characters, as well as definite and understandable enough to clarify regulatory mechanisms among the network of lipid metabolites–enzymes–genes.

Lipidomics methods are widely used in identification and validation of disease-specific biomarkers, which are required more sensitive and specific to disease severity, duration, subtype, and response to therapy. The diversity of lipids in healthy and diseases requires reliable methodologies for qualitative and quantitative detection of lipidomic profiles. Lipidomics is developing rapidly with the improvement of a strong sensitivity and precision in those advanced technologies. It is expected that more and more lipid ligands can be discovered in coming studies. The study and analysis methods of lipidomics are mainly based on mass spectrometry, e.g. liquid-chromatography or matrix assisted laser desorption/ionization mass spectrometry imaging. Those methodologies have the ability to analyze a large of lipids with different physico-chemical properties, including polarity, solubility, and molecular weights [12]. For example lipid markers can be used in the diagnosis of non small cell lung cancer as a noninvasive biomarker. Biostatistical analysis on lipids can produce a highly sensitive and specifically predictive model to classify breast cancer patients between benign and malignant [13]. After comprehensive data analysis, lipid involved in glycerolipids, glycerophospholipids and sphingolipids metabolism especially phosphatidylglycerol (34:0), sphingomyelin (42:2), ceramide (44:5), lysophosphatidylcholine (18:3), sphatidylcholine (18:2), phosphatidylethanolamine (O-36:3), phosphatidylethanolamine (O-38:3) and sphingomyelin (38:8) were significantly proposed to be a potential biomarkers in colorectal cancer and showed an excellent specificity between healthy controls and colorectal cancer patients [14].

The study about lipids as biomarkers for cancer is gradually translated from basic research to clinical application. Phosphatidylethanolamine, ether-linked phosphatidylethanolamine and ether-linked phosphatidylcholine were considered as biomarkers in prostate cancer [15]. Recently study demonstrated that 1 mL of patient urine could be used to extract many different lipid species, and further utilized for diagnose of breast cancer [16]. An aberrant lipid metabolism was noticed in prostate cancer carcinogenesis and progression. The dysfunction of lipids, e.g. up-regulated lipid abundance and re-programming of lipid composition, was driven by enhanced lipogenesis, lipid uptake and phospholipids remodeling. Lipidomics is widely used to discover new therapies for cancer or provide a new trend of drug discovery and development. Drug resistance is a huge challenge on cancer drug therapies. Metabolomics of lipids are taken part in the resistance mechanism of some anticancer drug therapies. Phosphorylcholine could increase in drug sensitive cancer cells, and reduce in drug resistant cells. Cholesterol and diacylglycerols were treatment markers in hypoxic regions of cancer. Lipids is an important component of cell membranes which are interacted by drug firstly when triggering tumor cell death. Lipids metabolism of anticancer therapeutics can be one of the most important changes in patient with cancer. Lipids also modulate protein function and structure by directly or indirectly connecting with protein [17]. The complexes of milk protein α-lactalbumin and oleic acid have strongly potent tumoricidal and anti-microbial abilities. Lipid rafts are membrane microdomains and play important role in life cycle of microbes, responsible for the initial colonization or induction of inflammation. Baqam et al. proposed new intervention strategies to modulate the assembly of membrane rafts and/or regulate raft-directed signaling pathways as efficient approaches for infectious diseases [18].

Lipids have complex chemical structures which decide its diverse physiochemical properties, which make enormous challenges for clinical application of lipidomics [19]. The lipidomics-based studies have a promoting progress and future in biomarker identification, mechanisms exploration and therapeutic improvement of diseases. Lipids metabolism and its underlying regulation play critical roles in the development and progression of diseases. Lipidomics will provide new insights on molecular mechanisms and understanding of lipids in cancer processes and promote clinical applications in anticancer drug discovery and personalized therapy for diseases.
